# Personalized monitoring of drug therapy effectiveness based on high-throughput analysis of drugs utilization and inflammatory factors closed functional networks in cardiovascular pathogenesis pathologies

**DOI:** 10.1186/1878-5085-5-S1-A80

**Published:** 2014-02-11

**Authors:** Svetlana Babak, Larisa Litvinova, Maksim Patrushev

**Affiliations:** 1 Laboratory of Clinical Pharmacology, Immanuel Kant Baltic Federal University, 14, Nevskogo, Kaliningrad, 236041, Russia; 2 Laboratory of Immunology, Immanuel Kant Baltic Federal University, 14, Nevskogo, Kaliningrad, 236041, Russia; 3 Genome and Proteome Research laboratory, Immanuel Kant Baltic Federal University, 14, Nevskogo, Kaliningrad, 236041, Russia

## Objective

Personalized dynamic monitoring system development of a patient with cardiovascular disease on the basis of large-scale physical, genomic, proteomics, metabolomics, and immunological studies.

## Technical approaches

1. Remote real-time functional monitoring of patients;

2. Genome profiling (NGS);

3. Proteome profiling (IEF, LC MS/MS);

4. Cell function research (Flow cytometry, Live cell imaging);

5. Drugs and metabolites concentration control and association it with genomic constitution (LC MS/MS);

6. Quantification plasma concentration of the drugs and their metabolites, enzymes (LC MS/MS);

7. Immunologic profiling (ELISA, flow cytometry).

## Results

We examined patients –non-responding on pharmacotherapy. All patients had “wild genotype” of the isoenzymes of the cytochrome P450 [1]. Drug plasma level decreased in presence high activity of the vessel endothelium growth factor and interleukin – 8. NADPH –reductase activity has decreased also. These results are showing activity of inflammatory processes and progressive of the diseases [2]. However, expression gene of the p-glycoprotein is increased; PEPT transport activity is increased too. Genomics and proteomic monitoring is necessary in this case.

The effectiveness of drug treatment depends on the patient's genetic constitution. One of the general networks is the drugs utilization system, including proteins of transport and biotransformation system. Identification of polymorphisms in genes encoding proteins of these systems, as well as polymorphisms in genes of responsible for inflammatory and oxidative processes, provide a full-scale monitoring of the majority of the factors responsible for the effectiveness of drug therapy. In this case, monitoring of genetic factors in the personalized approach coupled with the assessment of immune system cells functioning, and general functional monitoring of patients (ECG, blood pressure), and a daily quantification relevant metabolites. The data based on the physiological parameters, the functional status of the immune system, genetic polymorphisms, and local protein profiles are compared with data on the pharmacokinetics compared with different therapeutic protocols. The result revealed fundamental laws of individual processes at different levels that take place in the treatment of cardiovascular disorders.

## Outlook for experts

Obtained data focused on the basic monitoring protocol development and identification of the new biomarkers. It is the first case of high throughout testing of different treatment protocols. Their comparison leads to developing of new treatment approaches based on individual molecular, cell and physiological parameters.
